# Parenteral vaccination protects against transcervical infection with *Chlamydia trachomatis* and generate tissue-resident T cells post-challenge

**DOI:** 10.1038/s41541-020-0157-x

**Published:** 2020-01-23

**Authors:** Nina Dieu Nhien Tran Nguyen, Anja W. Olsen, Emma Lorenzen, Peter Andersen, Malene Hvid, Frank Follmann, Jes Dietrich

**Affiliations:** 1 Statens Serum Institut, Department for Infectious Disease Immunology, Copenhagen, Denmark; 2 Department of Biomedicine and Department of Clinical Medicine, Aarhus University, Aarhus, Denmark

**Keywords:** Infectious diseases, Inflammation, Vaccines, Urogenital diseases

## Abstract

The optimal protective immunity against *Chlamydia trachomatis* (*C.t.)* is still not fully resolved. One of the unresolved issues concerns the importance of resident immunity, since a recent study showed that optimal protection against a transcervical (TC) infection required genital tissue-resident memory T cells. An important question in the *Chlamydia* field is therefore if a parenteral vaccine strategy, inducing only circulating immunity primed at a nonmucosal site, should be pursued by *Chlamydia* vaccine developers. To address this question we studied the protective efficacy of a parenteral *Chlamydia* vaccine, formulated in the Th1/Th17 T cell-inducing adjuvant CAF01. We found that a parenteral vaccination induced significant protection against a TC infection and against development of chronic pathology. Protection correlated with rapid recruitment of Th1/Th17 T cells to the genital tract (GT), which efficiently prevented infection-driven generation of low quality Th1 or Th17 T cells, and instead maintained a pool of high quality multifunctional Th1/Th17 T cells in the GT throughout the infection. After clearance of the infection, a pool of these cells settled in the GT as tissue-resident Th1 and Th17 cells expressing CD69 but not CD103, CD49d, or CCR7, where they responded rapidly to a reinfection. These results show that a nonmucosal parenteral strategy inducing Th1 and Th17 T cells mediates protection against both infection with *C.t*. as well as development of chronic pathology, and lead to post-challenge protective tissue-resident memory immunity in the genital tract.

## Introduction

*Chlamydia trachomatis (C.t.)* is globally the most common sexually transmitted bacterium with an estimated 131 million new cases occurring every year.^1^ *C.t*. is an obligate intracellular bacterium infecting both men and women. Infections are frequently asymptomatic and consequently left untreated. Untreated women can experience serious sequelae such as pelvic inflammatory disease that can lead to fatal ectopic pregnancy and infertility.^2–4^ Preventing diseases caused by this pathogen require better understanding of the interaction between *C.t*. and the host.

Interactions between *C.t*. and host immune responses have, however, been difficult to examine due to lack of a model that can recapitulate a human *C.t*. infection and its disease progress. Mouse models in which *C.t*. is inoculated intravaginally efficiently infects the lower genital tract but generally do not lead to a robust infection in the upper genital tract and consequently this model does not develop the inflammation and pathology observed in humans.^5,6^ However, recently it was shown that by bypassing the cervix and inoculating *C.t*. directly in the upper genital tract (uGT), an efficient colonization of the uGT, was accomplished, which coincided with a robust inflammatory response.^6–8^ Moreover, reduced fertility and inflammation extending along the uterine horns was also observed, as well as histopathological changes including fluid buildup, infiltration of macrophages and neutrophils in uGT tissue and lumen.^6,8^ These findings suggest that the ‘transcervical’ (TC) infection model can be used to test the protective capacity of vaccines against both the infection as well as immunopathology.

CD4 T cells are known to play a protective role during a *C.t*. infection in mice, and specifically IFNγ-producing CD4 T helper-1 cells (Th1) have shown to be essential for protection.^6,9^ In line with the animal studies, *C.t*.-specific CD4 T cell responses is associated with protection against *Chlamydia* reinfection in women.^10,11^ Th17 T cells have also been observed during the course of a *C.t*. infection.^12^ However, further studies of Th17 T cells are required to determine their role, in particular as previous studies suggested that this particular subset of T cells may induce pathology rather than protect against it.^12–20^

Although a parenteral vaccine has proven effective against a vaginal infection with *C.t*.^21–23^ or *C.m*.,^24–30^ in the TC *C.t*. model, it was recently shown that mucosal primed (intrauterine or intranasal) vaccines are superior in achieving protection, due to induction of T cells that seed the uterine mucosa and remain as Trm cells that respond rapidly to infection.^7^ A parenteral vaccine administered at a nonmucosal site, induced circulating T cells that did not protect against a transcervical infection, in contrast to circulating T cells arising from a mucocal vaccine which induced partial protection. Thus, to which degree Trm cells are absolutely required for protection against infection, or if circulating immunity induced by a nonmucosal parenteral vaccine is sufficient, is not fully resolved yet. This is an important question as it is generally agreed that a parenteral vaccine represents the most safe administration form.

In the present study, the aim was therefore to examine the cellular recruitment of circulating CD4 cells generated by a nonmucosal parenteral vaccine, as well as the protective efficacy of such a vaccine. The vaccine consisted of the *C.t*. vaccine antigen CTH522,^31,32^ formulated in the adjuvant CAF01 (cationic adjuvant formulation 01), which possess a unique property of inducing circulating Th1 T cells as well as Th17 T cells.^33^ The data showed that a nonmucosal parenteral vaccine induced systemic immunity able to protect against a TC infection. Moreover, following an infection some of the recruited vaccine-induced T cells settled in the genital tract tissue as Trm cells, and upon a reinfection these cells responded rapidly to the infection.

## Results

### No protection against uGT infection with high infectious doses of 10^5^ or 10^6^ IFU, despite early recruitment of Th1 and Th17 T cells to the GT

**Fig. 1:**
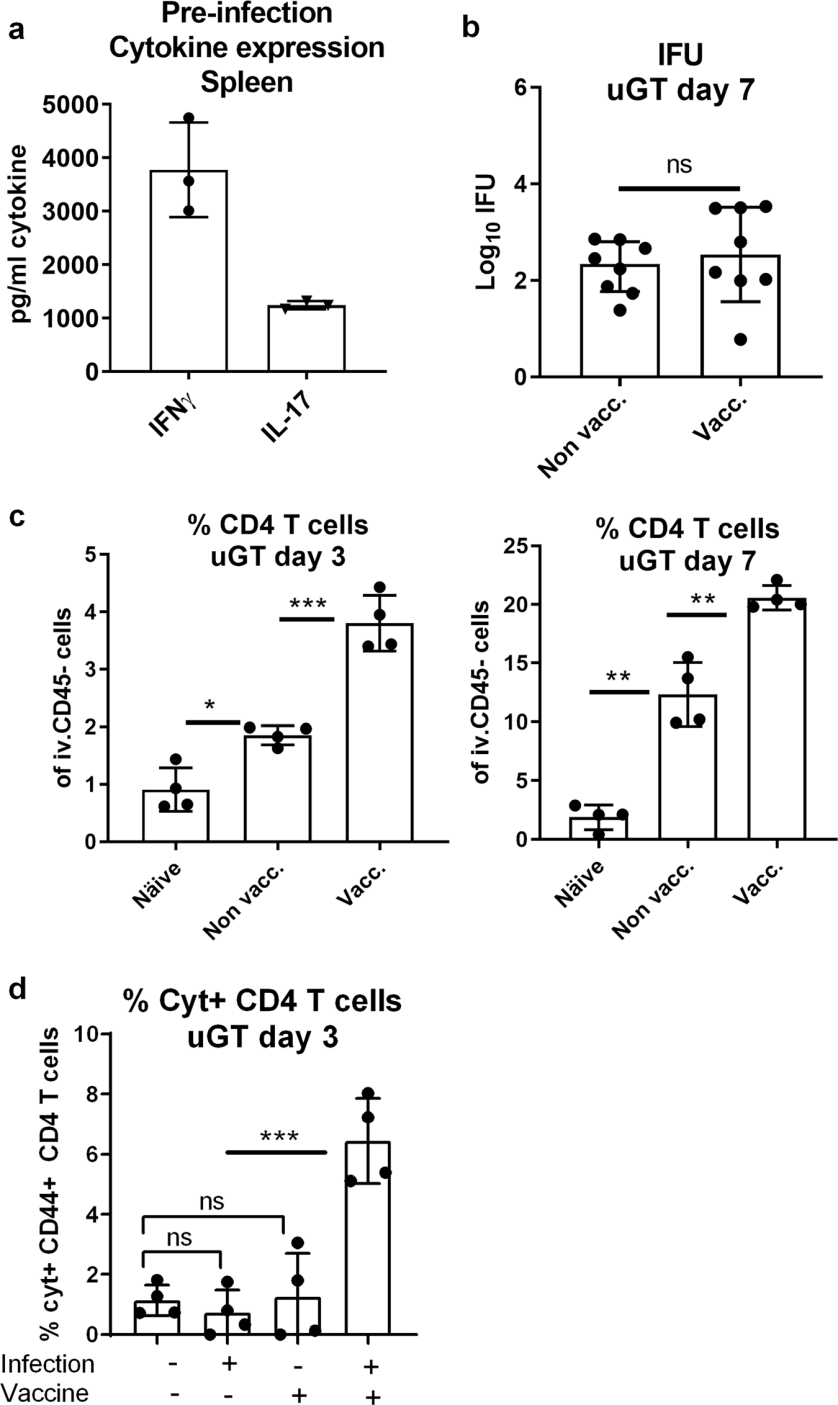
Protection against infection with 10^5^ IFU of *C.t*. SvD. Groups of female B6C3F1 mice (*n* = 8, pooled either pairwise or as 3 + 3 + 2 animals) were vaccinated three times s.c. with CTH522 antigen and CAF01 adjuvant, with two weeks intervals. Three weeks after the last vaccination, the mice received a TC infection with a 10^5^ IFU of *C.t*. SvD. Secretion of IFNγ and IL-17 from splenocytes were detected by MSD after in vitro stimulation with CTH522 (**a**). Bacterial burden in the uGT was measured by IFU count in cultured swab samples. The data are presented as Log10 of IFUs. Bars indicate medians with interquartile ranges (IQR) (**b**). 3 and 7 days post infection percentages of CD4+ T cells in uGT were determined by flow cytometry (**c**). Among the CD4+ T cells, percentages of cytokine (IFNγ, IL-2, IL-17, and/or TNFα) positive antigen-specific CD44+ CD4+ T cells were determined (**d**). Bars indicate means ± SD, *p < 0.05, **p < 0.01, ***p < 0.001, ns not significantly different.

We first examined if circulating immunity, induced by CAF01, was able to protect against a TC infection with *C.t*. Serovar D (SvD). We used an infection dose of 10^5^ or 10^6^ IFUs, as these doses were also used in other studies using the TC model.^6–8^ For the vaccine antigen we chose CTH522, which has previously been shown to protect against a vaginal infection with *C.t*.^31^ and to generate both Th1 and Th17 T cells.^27,34^ Mice were vaccinated three times, at two weeks intervals with CTH522/CAF01. To analyze the systemic T cell response, splenocytes were stimulated in vitro with CTH522 and secretion of IFNγ and IL-17 was analyzed (Fig. 1a). The results showed that cells from vaccinated animals stimulated in vitro with CTH522 released both IFNγ and IL-17, in agreement with previous studies.^35^ Six weeks following the last immunization, the animals were subjected to a TC infection and bacterial levels were determined in the uterus (upper genital tract, uGT) at day 7 post infection. The results showed that following infection with 10^5^ IFUs the vaccine did not reduce the bacterial numbers significantly (Fig. 1b). We also tested a dose of 10^6^ IFUs, and again we did not see any vaccine-induced protection (Supplementary Fig. 1a).

As slow or inefficient recruitment of circulating T cells might explain the lack of protection,^7^ we next examined how fast the vaccine-induced circulating immune cells were recruited to the genital tract post infection. To measure leucocytes in the genital tract tissue, and not intravascular leucocytes, mice were subjected to in vivo intravascular staining by injecting fluorescein isothiocyanate (FITC)-labeled anti-CD45 monoclonal antibody (mAb) intravenously (iv.CD45) 3 min before the mice were euthanized. This selectively stains intravascular, but not tissue lymphocytes (as described by Anderson et al,^36^) and enable us to exclude intravascular cells from the subsequent flow cytometry analysis (gating strategies for flow cytometry analyses are shown in Supplementary Figs 2 and 3). We first analyzed the percentage of CD4 positive T cells out of all iv.CD45-negative cells in the uGT. Already at day three post infection 3.81% of all cells in the uGT were CD4 T cells, which was more than observed in nonvaccinated infected mice (1.85% of all cells) (Fig. 1c). At day 7 post infection this number had increased to 20.58% of all cells. At day three 6.44% of all CD4 cells were cytokine-positive (IFNγ, IL-2, IL-17, or TNFα), i.e. antigen-specific. In contrast, in vaccinated (and noninfected) or infected (and nonvaccinated) animals, we did not detect vaccine specific T cells in the uGT (Fig. 1d). The increased percentage of CD4 T cells in the GT of vaccinated/infected mice was also reflected in the total number of CD4 T cells in the GT, which increased from day 3–7, whereafter it started to plateau (Supplementary Fig. 1b and c that show the actual number of cytokine-positive CD4 T cells in the GT).

These results demonstrated that the infection resulted in the recruitment of circulating Th1 and Th17 T cells very early after infection. However, despite this, no significant effect on the bacterial levels was observed.

### Protection against a transcervical infection with 10^3^ IFUs

**Fig. 2:**
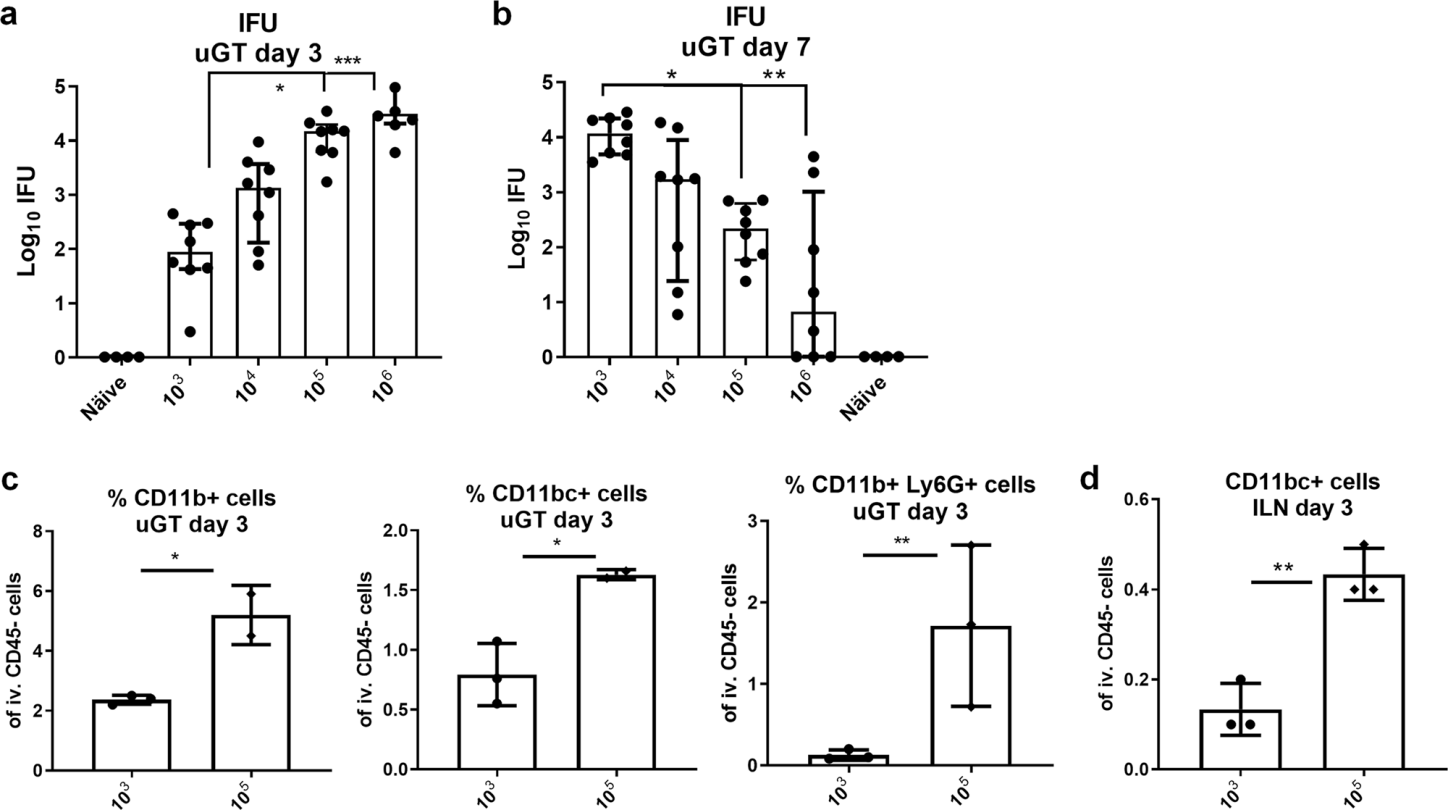
Testing different infection doses. Groups of female B6C3F1 mice (*n* = 8) were TC infected with different doses of *C.t*. SvD ranging from 10^3^ to 10^6^ IFU of *C.t*. SvD. 3 days and 7 days post infection, the bacterial burden in the uGT were determined in cultured swab samples. The data are presented as Log10 of IFUs. Bars indicate medians with IQR. Statistical significance was evaluated by a Kruskal-Wallis test followed by Dunn’s multiple comparisons test using GraphPad Prism version 7.04. (*p < 0.05, **p < 0.01) (**a**, **b**). Percentages of neutrophils (Ly6G^+^CD11b^+^), macrophages (Ly6G^−^CD11b^+^CD11c^−^) and dendritic cells (Ly6G^−^CD11b^+^CD11c^+^) were determined in the uGT and ILN 3 days post infection by flow cytometry. *n* = 8, animals were, pooled pairwise (**c**, **d**). Bars indicate means ± SD. Statistical significance was evaluated by an unpaired t-test using GraphPad Prism version 7.04. *p < 0.05, **p < 0.01.

To further explore the ability of a parenteral vaccine to protect against a TC infection, we next tested different infectious doses. One aim was to reduce the infection-induced innate immunity observed following infection with 10^5^ IFUs (Supplementary Fig. 4) as this can be protective in itself, making it difficult to measure the protection mediated by vaccine-induced adaptive immunity. At day 3 post infection we observed a clear IFU dose titration effect in the upper genital tract with significant differences between the dose of 10^3^ and 10^5^ or 10^6^ IFUs (Fig. 2a). However, at day 7 post infection the picture was reversed. Animals infected with 10^3^ IFUs now showed significantly higher bacterial numbers compared to animals infected with 10^5^ or 10^6^ IFUs that showed a decline in bacterial levels from day 3–7 (Fig. 2b). As expected, this correlated with a reduced percentage of both neutrophiles, monocytes, dendritic cells in the uGT in the animals receiving the 10^3^ IFU dose compared to animals receiving higher doses (Fig. 2c) at day 3 post infection. In the draining iliac lymph-node (ILN) mice infected with only 10^3^ IFUs also showed reduced recruitment of dendritic cells compared to mice infected with 10^5^ IFUs (Fig. 2d).

We also tested inoculation with doses of 10^2^ and 10^1^ IFUs (Supplementary Fig. 5). We did observe an infection of 71% of the animals with as little as 10 IFUs, and 88% infected animals with only 100 IFUs, but we chose to proceed with a dose of 10^3^ IFUs to protection studies, as this gave a 100% infection rate, reduced innate activation, and an increase in bacterial numbers from day 3–7 post infection instead of a decrease as observed when infecting with 10^5^ IFUs or more.

**Fig. 3:**
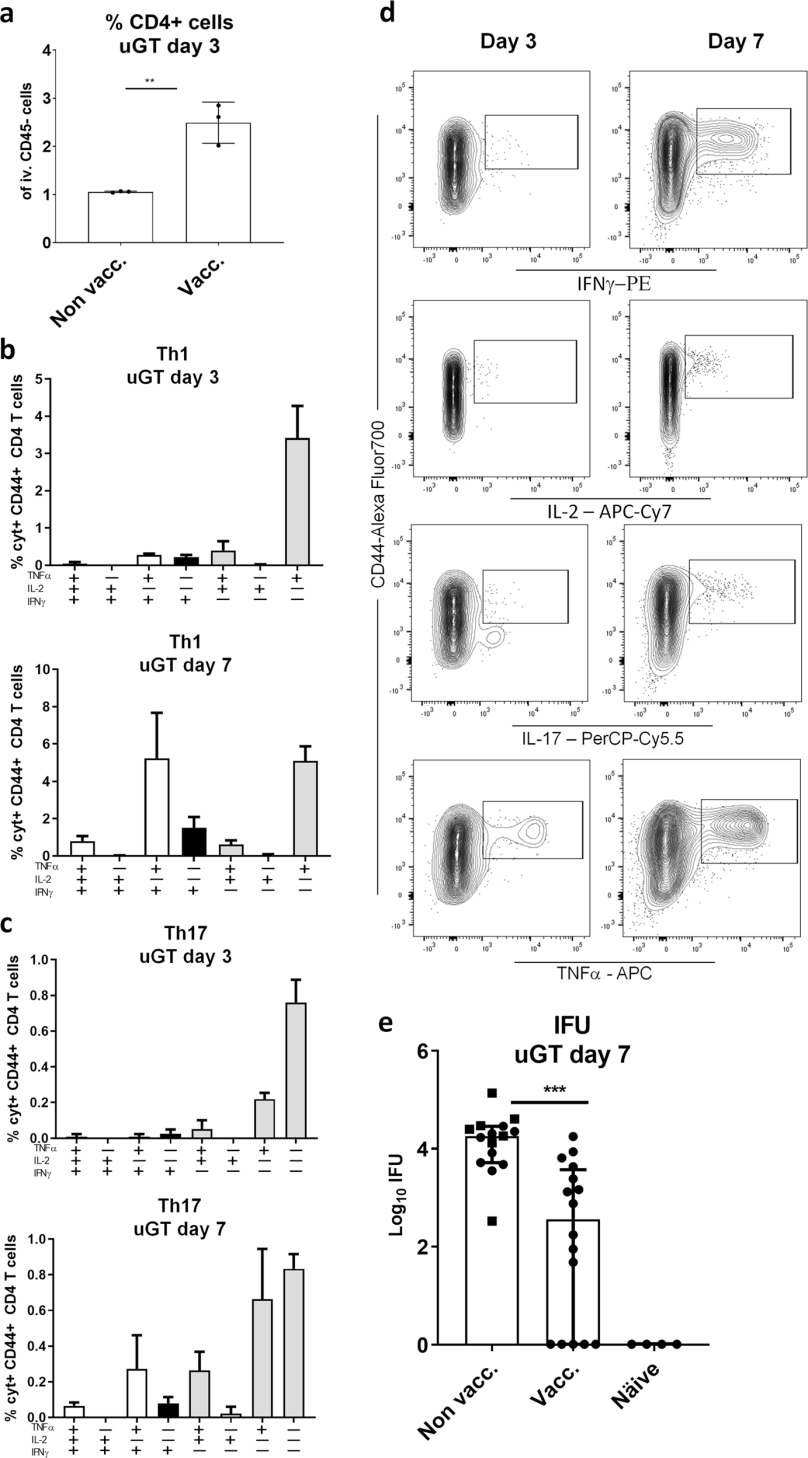
Protection against infection with 10^3^ IFUs of *C.t*. SvD. Female B6C3F1 mice (*n* = 8) were vaccinated three times s.c. with CTH522 antigen and CAF01 adjuvant, with two weeks intervals. Three weeks post immunization the mice received a TC infection with 10^3^ IFU of *C.t*. SvD. The percentages of CD4 + T cells in uGT at day 3 post infection, was analyzed by flow cytometry (animals were pooled as 3 + 3 + 2). Bars indicate means ± SD. Statistical significance was evaluated by an unpaired *t*-test using GraphPad Prism version 7.04. ****p < 0.0001 (**a**). Three and 7 days post infection percentages of IL-17 negative CD4 T cells (Th1) and IL-17 positive CD4 cells (Th17) out of all CD4+ T cells in the uGT were analyzed by flow cytometry for the frequency of cytokine subsets (expression of TNFα, IL-2, IFNγ and/or IL-17). Gray bars indicate resting T cell subsets (expressing IL2 and/or TNFα), white bars indicate effector subsets that also express INFγ and black bars indicate subsets expressing only IFNγ within the Th1 and Th17 population (**b**, **c**). Representative contour plots of the cytokine expressions (**d**). Bacterial burdens in nonvaccinated, vaccinated and näive female B6C3F1 mice (*n* = 16) were determined in cultured swab samples (**e**). Bars indicate medians with IQR. Statistical significance was evaluated by a Kruskal-Wallis test followed by Dunn’s multiple comparisons test using Graphpad Prism version 7.04. ***p < 0.001 (**e**).

Mice were vaccinated three times with CTH522/CAF01 as described above and infected transcervically with 10^3^ IFUs. At day 3 post infection we observed increased percentage of CD4 T cells(Fig. 3a). Both Th1 and Th17 T cells expressed TNFα and IL-2, but then progressed to also express IFNγ at day 7 post infection (Fig. 3b–d, or Supplementary Fig. 6, which show day 14 post infection in both vaccinated and nonvaccinated mice having received a dose of 10^3^, or even 10^2^ IFUs). In the figure, the different Th1/Th17 cytokine subsets^37^ are indicated by color. Gray bars indicate resting subsets (expressing IL2 and/or TNFα), white bars indicate effector subsets that also express INFγ and black bars indicate subsets expressing only IFNγ within the Th1 and Th17 population. Thus, also following a dose of 10^3^ IFUs, Th1, and Th17 T cells were rapidly recruited to the GT where they responded to the infection by changing their cytokine expression. However, compared to infection-driven Th1 and Th17 T cells in the GT of nonvaccinated animals, the Th1/Th17 T cells observed in vaccinated animals were of a significantly higher quality (Supplementary Fig. 6a). In fact, it was striking that the dominant cytokine profile among Th17 and in particular Th1 T cells in nonvaccinated animals were IFNγ^+^ cells expressing neither TNFα or IL2. Importantly, the reduced infection dose of 10^3^ IFUs enabled the parenteral CTH522/CAF01 vaccine to show protective efficacy. The bacterial numbers determined at day 7 post infection showed a significant reduction in the vaccinated animals from 4.26 to 2.56 Log10 IFU corresponding to a reduction in bacterial numbers of 98% (Fig. 3e).

Taken together, by infecting with a dose of 10^3^ IFUs we were able to show that circulating T cells can be subject to a fast recruitment to the GT, correlating with protection against a TCinfection with *C.t*.

### The fate of the recruited Th1 and Th17 cells in the genital tract

Having shown that circulating Th1 and Th17 T cells were recruited to the uGT early after infection, and that they responded to the infection by changing their cytokine expression, we next analyzed the fate of these i.v CD45-negative Th1/Th17 T cells in the uGT after the infection had been cleared. The objective was to determine to which degree some of the recruited cells from circulation settled in the GT as resident T cells.

**Fig. 4:**
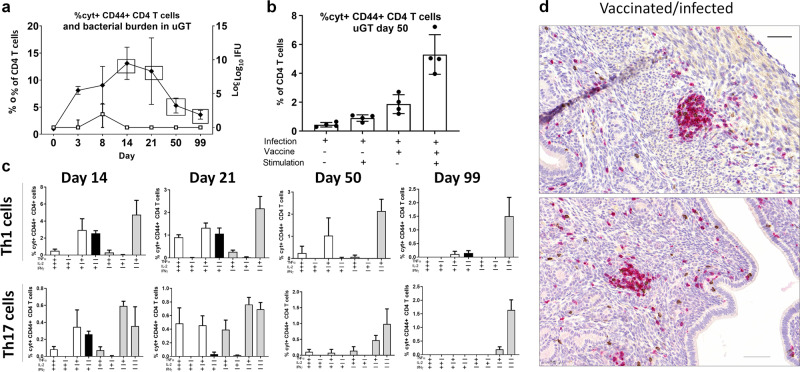
The fate of T cells recruited to the genital tract. Groups of female B6C3F1 mice (*n* = 8) were vaccinated three times s.c. with CTH522 antigen and CAF01 adjuvant, with two weeks intervals. 3 weeks post immunization the mice received a TC infection with 10^3^ IFU of *C.t*. SvD. Percentages Cyt + CD44 + CD4 T cells out of all CD4 + T cells were determined by flow cytometry (closed circle) at the indicated days post infection (animals were pooled pairwise). Points and error bars indicate means ± SD. The bacterial burden to the indicated days post infection were determined by cultured swab samples (open square). Points and error bars indicate medians with IQR (**a**). At day 50 post infection, percentages of cyt + CD44 + CD4 + T cells in uGT of nonvaccinated and vaccinated mice were evaluated with and without CTH522 antigen re-stimulation. Bars indicate means ± SD (**b**). Percentages of cytokine subsets of IL-17 negative CD4 cells (Th1) and IL-17 positive CD4 cells (Th17) out of all CD4 + T cells in uGT were determined by flow cytometry at the indicated days post infection. Gray bars indicate resting T cell subsets (expressing IL2 and/or TNFα), white bars indicate effector subsets that also express INFγ and black bars indicate subsets expressing only IFNγ within the Th1 and Th17 population. Bars indicate means ± SD (**c**). At day 50 post infection genital tracts (GT) were removed, fixed and sectioned for immunohistochemical staining of CD4 (Permanent red chromogen). Shown is two representative images (20 X magnification) of a uterine horn section from a vaccinated/infected mouse (*n* = 8). Scale bar: 50 µm (**d**).

Vaccinated animals were infected and IFU levels were determined in the uGT. The results showed that IFU levels increased until day 7 post infection, whereafter they declined to background levels at day 14 post infection. Following the clearance of the bacteria the CD4 T cell subtypes were analysed in the uGT. At day 14 the percentage of antigen-specific cytokine-positive CD4 T cells (expressing either IFNγ, IL-2, IL-17, or TNFα) constituted 13% of all CD4 T cells in the uGT. This number declined to 11% at day 21 and 3% at day 50 post infection (Fig. 4a). The percentage of CD4 T cells also peaked around day 14 post infection (Supplementary Fig. 7). As expected, at day 50 or 99 post infection we could only stimulate cytokine expression in uGT CD4 cells taken from both vaccinated/infected animals (Fig. 4b).

Analysis of the cytokine subsets in the uGT showed that the Th1 effector cytokine subsets expressing TNFα/IFNγ and TNFα/IFNγ/IL-2 were present at day 14 and day 21 post infection. However, at day 50 and 99 post infection, these cytokine subsets were reduced resulting in cytokine subsets dominated by expression of TNFα (Fig. 4c). The same cytokine subset pattern was observed in the pool of T cells expressing IL-17 (Th17 T cells) (Fig. 4c), and it resembled the cytokine subsets observed at day 3 post infection (Fig. 3b, c).

To visualize the CD4 T cells in situ in the GT late after infection (day 50 post infection) we performed an immunohistochemical analysis. Vaccinated/infected mice were mostly dominated by CD4 T cell infiltrations, predominantly located in the subepithelial layers and often found in subepithelial clusters (Fig. 4d). Additional stainings from vaccinated and nonvaccinated mice are shown in Supplementary Figs 9–11.

Taken together, following clearance of the bacteria, a small pool of recruited Th1 and Th17 T cells, often located in subepithelial clusters, remained in genital tract as T cells showing a resting phenotype.

### Characterization of tissue-resident Th1/Th17 cells and their response to reinfection

**Fig. 5:**
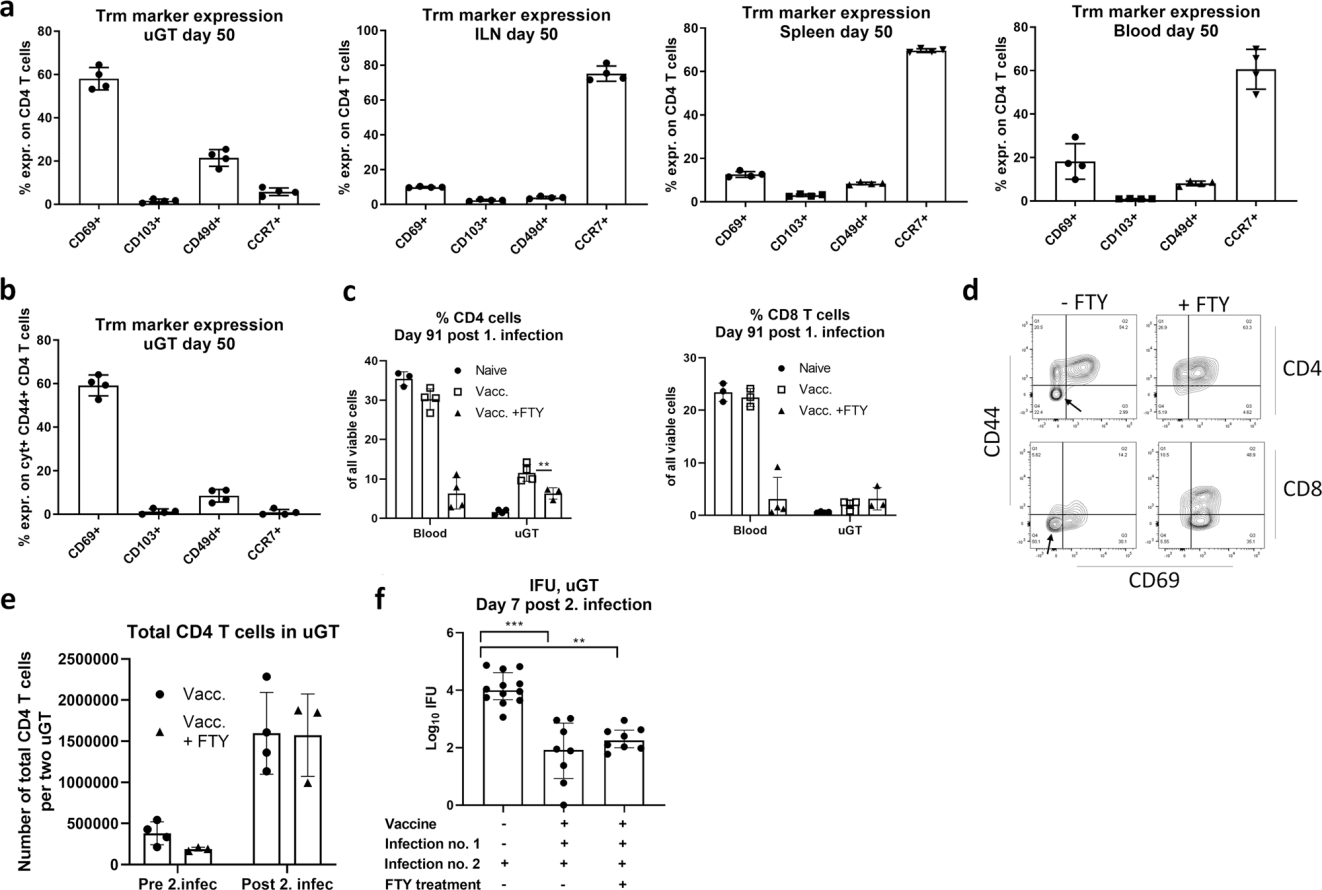
Characterization of resident memory T cells in the genital tract and their response to reinfection. Groups of female B6C3F1 mice (*n* = 8) were vaccinated three times s.c. with CTH522 antigen and CAF01 adjuvant, with two weeks intervals. 3 weeks post immunization the mice received a TC infection with 10^3^ IFU of *C.t*. SvD. 50 days post infection expression of CD69, CD103, CD49d and CCR7 were determined on CD4+ T cells in the uGT, ILN, spleen and blood (animals were pooled pairwise) (**a**). Expression of the same surface makers were determined on cyt+ CD44+ CD4+ T cells in the uGT at day 50 post infection (**b**). Groups of female B6C3F1 mice (*n* = 8) were vaccinated three times s.c. with CTH522 antigen and CAF01 adjuvant, with two weeks intervals. Three weeks after the last vaccination, the mice received a TC infection with a 10^3^ IFU of *C.t*. SvD. The animals were subjected to a reinfection 92 days post the primary infection. One group were treated with FTY720 to block T cell egress from the lymph-node and at day 91 post first infection % CD4 and CD8 T cells was examined in blood (**c**). Among the CD4 and CD8 T cells in the GT tissue, the percentages of CD44 and CD69 expressing cells were determined. The expression of CD44+/− and CD69+/− cells in the T cell population in the uGT are shown as a representative contour plot. A black arrow indicate the population of T cells particularely affected by the FTY treatment (**d**). On day 7 post 2. infection total number of CD4 and CD8 T cells in the uGT were determined (**e**). Bacterial burden in the uGT was measured by IFU count in cultured swab samples. A nonvaccinated control group receiving only the second infection was included. The data are presented as Log_10_ IFUs (**f**). Bars indicate medians with interquartile ranges (IQR). Bars in **c** and **e** indicate means ± SD, *p < 0.05, **p < 0.01, ***p < 0.001.

We next characterized the T cells in the uGT further in terms of surface markers. We decided to look at day 50 post infection. The vast majority of T cells in the uGT were CD44-positive indicative of antigen experienced effector/effector memory T cells. However, on CD4 T cells we did not observe a high expression of neither CD103 or CD49d (Fig. 5a). The receptor showing the highest expression was CD69. Expression of CCR7 was low in the GT but high in the draining LN (Fig. 5a). Analysis of cytokine-positive T cells showed the same pattern (Fig. 5b). Taken together, the pool of Trm cells in the uGT showed low expression of all the markers tested except for CD69.

We finally evaluated how these Trm cells responded to a reinfection, and if protection against reinfection was dependent on trafficking of antigen-stimulated T cells from the draining ILN to the GT (via the blood), or if the GT Trm cells were sufficient to provide initial protection. To do this mice posessing Trm cells in the GT were subjected to fingolimod (FTY720), which inhibit lymphocyte egress from lymph nodes and reduce the number of circulating peripheral lymphocytes. Mice having previously received a parenteral CTH522/CAF01 vaccine, as well as an infection, were divided into two groups, where one group received fingolimod (FTY720) in the drinking water daily from day 77 post the first infection and throughout the experiment. At day 92 post the first infection, the animals were reinfected and the immune response and bacterial levels in the uGT were examined at day 7 post the reinfection.

Before killing the mice, the mice received labeled anti-CD45 mAb i.v. and the uGT was analyzed. FTY treatment strongly reduced the lymphocytes in the blood (Fig. 5c). Some reduction in CD4 T cells (and no reduction in CD8 T cells) was observed in the GT (Fig. 5c). In particular one population was affected by FTY treatment, namely CD44^−^CD69^−^ T cells (most probably naïve T cells) (Fig. 5d). Importantly, both tissue Th1/Th17 cells were strongly expanded by the reinfection, irrespective of FTY treatment (Fig. 5e). Moreover, FTY treatment did not lead to increased bacterial numbers in the upper GT (Fig. 5f). Thus, the reduced lymphocyte numbers in circulation, as well as the lack of transport of lymphocytes from the ILN to the GT, did not compromize the T cell response in the GT, as well as protection against reinfection. This suggested that the Trm cells were capable of mediating protection against reinfection (Fig. 5f).

Taken together, circulating immunity induced by a nonmucosal parenteral vaccine protect against a TC infection and lead to resident T cells in the genital tract that respond rapidly to reinfection.

### Circulating immunity induced by a nonmucosal parenteral vaccine protect against chronic pathology in the genital tract

**Fig. 6:**
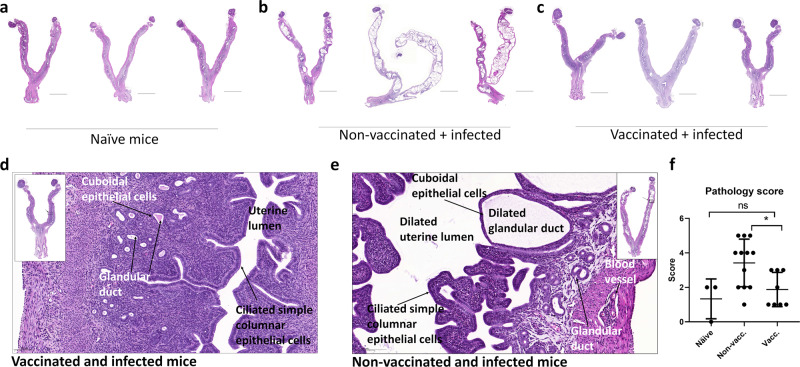
Histopathological changes post infection in vaccinated and nonvaccinated mice. Groups of female B6C3F1 mice (*n* = 8–12) were vaccinated three times s.c. with CTH522 antigen and CAF01 adjuvant, with 2 weeks intervals. Three weeks post immunization the mice received a TC infection with 10^3^ IFU of *C.t*. SvD. At day 50 post infection the genital tracts (GT) were removed, fixed and stained with hematoxylin and eosin (HE). Age-matched naive mice (*n* = 3) were used as controls. 0.4× magnification of representative GTs from naive mice (**a**), nonvaccinated mice (**b**) and vaccinated mice (**c**). Scale bar: 5000 µm. 5.0× magnification of uterine horns of vaccinated mice (**d**) and nonvaccinated mice (**e**), showing the tissue components. Scale bar: 100 µm. All animals were assigned a pathological score, based on the severity of the hydrometra and glandular duct dilation. Bars indicate means ± SD (**f**). Statistical significance was evaluated by an ANOVA test followed by Tukey’s multiple comparisons using Graphpad Prism 7.04. *p < 0.05, ns: not significant.

As the presence of Th17 T cells has been suggested to correlate with increased pathology in the upper genital tract in some studies,^12–16^ but not in others,^17–19^ it was important to also examine the effect of the recruited T cells on development of pathology. Mice were vaccinated as described above, and at day 50 post a TC infection with 10^3^ IFUs, the genital tract was analyzed for chronic pathological changes. The histological sections shown in Fig. 6 are examples of GTs from nonvaccinated + infected, vaccinated + infected, and age-matched naïve mice as a control. Scoring, based on the severity of the hydrometra and glandular duct dilation, of all mice is shown in Fig. 6f, and Supplementary Fig. 8 show examples of the different scoring values. The naïve mice showed uterine horn tissue with a well-defined small lumen and an intact endometrial layer with tubular structures that extends into the glandular ducts in the endometrial stroma. (Fig. 6a). In contrast, compared to the naïve mice, the H&E stained sections of the GT from the nonvaccinated/infected mice showed severe pathological changes giving a score of 4 or 5 in the uterine horns in 7 out of 12 mice (Fig. 6f). The primary pathological findings in infected mice were hydrometra (fluid filled, distended uterine lumen) and the glandular ducts appeared dilated compared to vaccinated + infected mice (Fig. 6d, e). In summary, nonvaccinated + infected mice showed a significantly higher pathology score compared to the vaccinated + infected group (Fig. 6f). No pathological changes were observed in the ovaries and oviducts from any of the animals.

## Discussion

It is critically important that a future *Chlamydia* vaccine is able to induce protective immunity that homes to the GT and protects against both infection and pathology. In the current study, we studied the recruitment of circulating Th1 and Th17 T cells to the GT following a transcervical infection, and if circulating immunity induced by a nonmucosal parenteral vaccine was sufficient to provide protection against both the infection and the development of immunopathology in the genital tract. Circulating immunity was induced by a parenteral vaccine consisting of CTH522 formulated in the adjuvant CAF01, which have been shown to induce protective immunity against vaginal infection with *C.t*.^31,34^

The result showed that protection against infection was dependent on the dose used. At a dose of 10^5^–10^6^ IFUs we did not observe any vaccine-induced protection, despite fast recruitment of circulating vaccine-induced Th1 and Th17 T cells into the uterine tissue, and a clear response of these cells to the infection (i.e., a change in their cytokine expression profile) (Fig. 1). However, reducing the dose to 10^3^ IFUs allowed for an increase in bacterial numbers from day 3 to day 7 post infection, instead of the decrease observed in the groups receiving 10^5^–10^6^ doses (Fig. 2), most probably due to a decreased activation of innate immunity in the GT when using the 10^3^ dose. Importantly, using this dose also allowed for a protective readout. In a previous study in the *C.m*-model. it was found that the lowest infection dose gave the highest bacterial load in the lower reproductive tract,^38^ and another study found that the ability of the bacteria to ascend to oviduct tissue was increased with reduced *C.m*. doses, which correlated with decreased innate immunity.^39^ Taken together, in models using vaginal infection with *C.m*. or TC infection with *C.t*. there are now several studies that highlight that choosing the correct dose is important, most probably because protection is a balance between natural-induced innate protective immunity and vaccine-induced adaptive protective immunity.

It should be noted that as the vaccine used in the present study also generate antibodies known to induce protection against a vaginal infection,^31^ the observed protection against a transcervical infection with 10^3^ may also involve antibodies. Experiments using mice deficient in producing antibodies or depleted for CD4 T cells or IL-17 are required to conclusively establish the protective role of the vaccine-generated CD4 Th1/Th17 T cells. These experiments are underway.

Vaccinated animals showed fast recruitment of circulating Th1/Th17 cells, which correlated with an increase in the percentage of neutrophils, macrophages and dendritic cells, and with protection (Fig. 3). This is in agreement with other studies showing that the cytokines IFNγ and IL-17 lead to recruitment and/or activation of innate cells.^13^ IL-17 has been shown to be important for establishing a Th1 response in a murine model of genital *Chlamydia* infection and in the *Herpes simplex virus 2* animal model.^13,19,20^ Whether IL-17 plays a similar role during a *C.t*. infection remains to be shown.

Our data show that Th17 T cells can be divided into several cytokine-producing subsets that is subject to a dynamic change during an infection. Other studies have also shown that Th17 cells can express IFNγ or even become ex-Th17 T cells that secrete only IFNγ,^40,41^ and such IFNγ-expressing Th17 subsets have been suggested to be associated with a pathological response. Interestingly, a previous study on group A streptococcus indicated that only infection-driven Th17 T cells, and not vaccine-induced Th17 T cells, were involved in development of a pathological response.^41^ In line with this, we show that the Th17 cytokine subsets in vaccinated/infected animals differ significantly from those observed in nonvaccinated/infected animals (Supplementary Fig. 6).

TNFα and IFNγ have been identified as mediators that contribute to the conditioning of the inflammatory site for high-rate accumulation of T effector cells.^42^ Our studies show that both Th1 and Th17 T cells, upon entering the GT, quickly change their cytokine expression pattern to include both TNFα and IFNγ. It will be important to demonstrate if Th17 T cells (and not only Th1 T cells) serve a specific role in delivering these effector cytokines to the inflamed environment.

Previous studies have implicated several receptors associated with either recruitment to the GT or retainment in the GT, i.e., establishment of a Trm cell population. CD103 has been observed on CD8 Trm cells.^43^ Its expression on CD4 T cells is still controversial, although some studies do report CD103 on CD4 Trm cells.^44^ Moreover, a recent study identified CD49d (α4) on CD4 T cells being recruited to the upper genital tract.^45^ CD69 has been observed on both CD4 and CD8 Trm cells.^44^ Our results showed that cells in the GT at day 50 post infection only expressed low levels of CD49d and CD103 (Fig. 5). In addition they showed low levels of CCR7, in contrast to the draining LN, in which up to 75% of all CD4 T cells expressed CCR7 (Fig. 5a). Furthermore, in the GT CD69 was expressed on up to 60% of the cyt + T cells (Fig. 5b). This indicates that establishment of resident T cells from T cells originating from circulation may not be strictly dependent on neither α4 or CD103, but does involve CD69 in agreement with previous studies on CD4 Trm cells in various tissues^46^ including the genital tract.^47^ Interestingly, our data showed that inhibiting lymphocyte egress from the lymph nodes, including the infection-draining lymph nodes, resulting in a strong decline in T cell numbers in blood, did not reduce the expansion of T cells in the GT or compromize the protection. This showed that in the first phase of the infection, the Trm cells were sufficient to induce a protective immune response (Fig. 5c–f).

As for the type of pathology observed, the tissue damage in the nonvaccinated/infected animals was confined to the uterine horns, including hydrometra and dilated glandular ducts, and no pathology was observed in the oviducts. This type of pathology was also observed in other recent studies.^6,48,49^ Pathology in the oviducts, more specifically hydrosalpinx, is a known pathological marker for infertility in mice. Others have, however, also observed a correlation between infertility and uterine horn and glandular duct dilation,^49^ and the TC model (using *C.t*.) have been reported to induce infertility in mice.^8^ Dilation of the glandular duct has also been observed in women with adenomyosis.^50,51^ A connection between adenomyosis and a *C.t*. infection remains to be shown, however.

The data showed that CTH522/CAF01 vaccination induced protection against these pathological changes. This is interesting since previous data suggested that Th17 T cells may have a role in inducing pathology.^12–20^ As the Th17 T cell cytokine profile differed markedly from the Th17 cytokine profile in nonvaccinated/infected animals (that developed pathology), it could be speculated that only certain Th17 T cell subsets are associated with pathology, and that a Th17-inducing vaccine can prevent induction of the pathology-inducing Th17 T cells that are normally induced by the infection itself.

In the present study, we present evidence that a parenteral vaccine, administered at a nonmucosal site, can induce circulating T cells that are rapidly recruited to the GT following infection, which in turn correlated with protection. Moreover, this also led to a population of Trm cells consisting of both Th1 and Th17 T cells that responded rapidly to a reinfection with *C.t*., and our data suggested that these Trm cells are indeed protective. Whether they are absolutely required, as have been suggested,^7^ is, however, another question, and may depend on the nature of the circulating immunity. In the present study we used a unique adjuvant system and antigen, which might explain why the Trm cells were not absolutely required to achieve protection. Although previous studies using a vaginal *C.m*. infection model showed that mucosal immunity added to the protection induced by a parenteral vaccine,^26^ a parenteral vaccine on its own did protect against infection with *C.m*.,^24–30^ even when an intrabursal challenge was used,^25^ again suggesting that Trm cells may not be absolutely required.

In summary, our data demonstrate that a nonmucosal parenteral vaccine can be sufficient in terms of protection against both the infection and pathology, and can lead to post infection resident T cells in the genital tract tissue that will respond rapidly to a reinfection. This is encouraging regarding future clinical trials with the CTH522/CAF01 vaccine, as well as other *Chlamydia* vaccines.

## Methods/experimental

### Ethics statement

Experiments were conducted in accordance with the regulations set forward by the Danish Ministry of Justice and animal protection committees by Danish Animal Experiments Inspectorate Permit 2018-15-0201-01502 and in compliance with European Community Directive 2010/63/EU of the European parliament and of the council of 22 September 2010 on the protection of animals used for scientific purposes, as well as Directive 86/609 and the U.S. Association for Laboratory Animal Care recommendations for the care and use of laboratory animals. The experiments were approved by a local animal protection committee at Statens Serum Institut, IACUC, headed by DVM Kristin Engelhart Illigen.

### Animals

Studies were performed with 6- to 8-week-old female B6C3F1 hybrid mice from Envigo, Scandinavia. Animals were housed in appropriate animal facilities at Statens Serum Institut and handled by authorized personnel.

### Bacteria preparations and transcervical infection

C.t. SvD (ATCC) were grown in HeLa cells (ATCC) in RPMI 1640 media (Invitrogen) supplemented with 1%HEPES, 1% of Nonessential amino acids (NEAA) (MP Biomedicals), 1% L-Glutamin (Gibco) and 1% pyruvate (Gibco). The infected HeLa cells were grown for 2–3 days at 37 °C at 5% CO_2_. Infected HeLa cells were harvested and *C.t*. were purified from the cells. Purified *C.t*. were resuspended in SPG buffer (250 mM Sucrose, 10 mM Na_2_HPO_4_, 5 mM L-glutamic acid) in aliquots at a concentration of 2.7*10^7^ IFUs/ml. Aliquots were stored at −80 °C

All mice were treated 10 and 3 days before infection with 50 mg of medroxyprogesteron to synchronize the murine eostrous cycle. Mice were transcervically infected using a thin, exible probe: Nonsurgical embryo transfer (NSET) device (Paratechs) to bypass the cervix and to inject bacteria directly into the uterine horn lumen.

### Antigens, adjuvant and immunization

Mice were immunized three times at 2-week intervals by the subcutaneous route (s.c.) (volume 200 µl) at the base of the tail. CAF01 was used as the adjuvant with a DDA/TDB dose of 250 µg/50 µg per mouse. The vaccine antigen used was a recombinant *Chlamydia* antigen designated CTH522,^31^ based on the MOMP (major outer membrane protein) protein. Two weeks after the last vaccination the immune response was analyzed.

### Fingolimod (FTY720) treatment

FTY720 (Sigma–Aldrich Denmark) was diluted in sterile 1xPBS to a concentration of 2 mg/L. The solution was administered ad libitum as the drinking water to the animals from 15 days before the second infection until day 7 post the second infection.

### Bacterial burden

To quantify the bacterial burden in the infected mice, swabs from the upper genital tract were collected. Swabsticks were cut and stored in 600 mL SPG buffer (250 mM Sucrose, 10 mM Na2HPO4, 5 mM L-glutamic acid) with plastic beads. The samples were stored at −80 °C. For cell passage, McCoy cells (ATCC) were seeded in infection media (RPMI 1640 (Invitrogen), 1% HEPES (Gibco), 3% infection supplement, 20% FBS, 0.18% Gentamicin (Gibco)) at a concentration of 0.16 × 10^6^ cells/ml in a 48-well plate (Costar) and incubated at 37 °C with 5% CO_2_ overnight. Cell media was aspirated and 0.2 ml glucose infection media (infection media 0.05% glucose) was added to the wells and incubated at 37 °C with 5% CO_2_. Undiluted and 1:2 diluted samples were added to the wells and incubated at 37 °C. Next, supernatants were aspirated and 0.5 mL glucose infection media with 1:1000 Cyclohexamid (Sigma) were added to the wells and incubated for one day at 37 °C. The cells were then fixated with 0.4 mL 96% ethanol per well. The cells were then dyed with 0.2 mL/well propidiumiodid (Sigma) (solution 1:2) and afterwards 0.25 mL/well of sterile-filtrated diluted rabbit anti-SerovarD MOMP antibody (in house) was added to label the inclusion bodies and subsequently incubated for 1 h at room temperature. Next the cells were incubated with Alexa Flour 488 conjugated secondary antibody goat anti-rabbit IgG (0.1 mL/well, Life Technologies) diluted 1:500 in 1× PBS 1% BSA. The plates were then incubated in the dark at room temperature for 1 h and afterwards kept at 4 °C. The IFUs per sample were quantified by fluorescence microscopy.

### Sample collection and cell preparation

Samples were obtained from eight mice per group and pooled either pairwise or in 3,3 and 2 organs per group in RPMI 1640 (Gibco Invitrogen). Three minutes before euthanasia, 250 µl of anti-CD45.2—fluorescein isothiocyanate (BD Pharmingen, clone 104, 1:100 dil.) were intravenously injected into the tail of the mice to label vascular leukocytes. Single-cell suspensions were created by homogenizing organs through a 100 µm nylon filter (Falcon). In addition. GTs were incubated before homogenization for 1 h at 37 °C CO_2_ in type IV collagenase (0.8 mg/ml) (Sigma), 30 min in DNAse I (Roche) (0.08 mg/ml). Before and after incubation GTs were processed with gentleMACS™ Dissociator (Miltenyi Biotec). Cell suspensions were centrifuged (700 × *g*, 5 min) and washed twice in RPMI 1640. Cell pellets were then resuspended in RPMI-1640 (Gibco Invitrogen) supplemented with 5 × 10^−5^ M 2-mercaptoethanol, 1 mM glutamine, 1% pyruvate, 1% penicillin-streptomycin, 1% HEPES, and 10% FCS (Gibco Invitrogen).

### Flow cytometry and biodistribution analysis

For intracellular cytokine staining, cells were stimulated for 1 h in the presence of CTH522 antigen and 1 µg/ml of costimulatory antibodies CD28 (BD Pharmingen, clone: 37.51) and CD49d (BD Pharmingen,clone: 9C10 (MFR4.B)). Brefeldin A was afterwards added at a concentration of 200 µg/ml to each sample and were subsequently incubated at 37 °C for 5 h and kept at 4 °C until staining. Cell suspensions were Fc-blocked with anti-CD16/CD32 antibody (BD Pharmingen, clone 2.4G2, 1:100 dil.) for 10 min. at 4 °C. Cells were stained with combinations of the following anti-mouse antibodies conjugated to fluorochromes (company, clone, dilution): α-CD4-BV786 (BD Horizon, GK 1.5, 1:600), α-CD44-Alexa fluor 700 (Biolegend, IM7, 1:150), α-CD8-BV421 (Biolegend, 53–6.7, 1:200), α-CD69-PE-Cy7 (BD Pharmingen, H1.2F3, 1:200), α-IL-2-APC-Cy7 (BD Pharmingen, JES6-5H4, 1:200), α-IFNγ-PE (BD Pharmingen, XMG1.2, 1:200), α-TNF-APC (BD Pharmingen, MP6-XT22, 1:200), α-IL-17-PerCP-Cy5.5 (Invitrogen, eBIO17B7, 45-7177-82, 1:200), α-CD11b-APC-Cy7 (Biolegend, M1/70, 1:100), α-CD11c-APC (BD Pharmingen, HL3, 1:100), α-Ly6G-PE (BD Pharmingen, HL3, 1:100), α-CCR7-PE-Cy7 (Invitrogen, 4B12, 1:25), α-CD49d-BV421 (BD Optibuild, R1-2, 1:200), α-CD103-BV421 (BD Optibuild, M290, 1:200), and α-Viability-eFluor506 (Invitrogen, catalog number: 65-0866-14, lot: 2010926, 1:500).

The stained cells were analyzed using a Flow cytometer (BD LSRFortessa, BD Bioscience) and FlowJo Software (version 10). To analyse the cells in the organs we excluded, from the datasets, doublets on forward scatter height (FSC-H) and FSC area (FSC-A) plot and excluded cell debris on side scatter height (SSC-H) and FSC-H. These gated cells were designated as “all the cells” in the results. Leukocytes were divided into CD4 T cells (CD4^+^), CD8 T cells (CD8^+^), neutrophils (Ly6G^+^ CD11b^+^), macrophages/monocytes (Ly6G^−^CD11b^+^CD11c^−^) and dendritic cells (Ly6G^−^CD11b^+^CD11c).

### MSD analysis

MSD U-plex was performed to quantify levels of IFNγ and IL-17 expression. Harvested supernatants from CTH522 stimulated cells (stimulated for 72 h) were diluted 1:4 and added to 96-well multi-SPOT plate (MSD). The cytokine levels were quantified according to the manufactor’s instructions. The standard and samples were measured in duplicate and blank values were subtracted from all readings. The plates were read and analysed by SECTOR® Imager (MSD).

### Histological and immunohistochemical staining and analysis

Genital tracts from the mice were removed following euthanasia and fixed at room temperature in 4% formaldehyde (VWR chemicals) and paraffin embedded.

Processing, sectioning and staining were done by the technical staff at BioSiteHisto (Finland). Four micrometer thick sections were collected on Superfrost Plus slides (Thermo Fischer) and stained with hematoxylin and eosin (H&E). Furthermore 4 µm thick sections were collected on TOMO adhesive coated slides for immunohistochemical (IHC) staining. Before IHC staining, epitope retrieval was performed as described previously.^52^ The IHC staining method was based on peroxidase and phosphatase reactions to detect CD8 and CD4 epitopes. Detection of CD8 was performed with a rabbit anti CD8 alpha monoclonal antibody (Abcam, ab209775), an HRP based detection system and a brown DAB chromogen. CD4 was detected with a rabbit-anti-CD4 monoclonal antibody (SinoBiological, 50134-R001) and an AP based detection system and a permanent red chromogen. The IHC slides were counterstained with Mayer Hematoxylin (Merck). Slides were digitalized as WSI in Mirax format with 3DHistech Panoramic MIDI scanner (3DHistech Ltd.). Slides were visualized and analyzed using Caseviewer version 2.3.

### Pathology score

H&E stained tissue sections were assigned an inflammatory score according to the degree of hydrometra/uterine lumen dilation and glandular duct dilation. The following scores were used: 0; no dilation, 1; very mild dilation, 2; mild dilation, 3; moderate dilation with glandular duct dilation, 4; moderately severe dilation and moderate glandular duct dilation, 5; severe dilation and severe glandular duct dilation (pictures showing the different scores are shown in Supplementary Fig. 6). Tissue sections immunohistochemically stained for CD4 and CD8 were assigned a cell infiltration score for CD4 and CD8 cells separately. The following scores were used: 0; very few cells, sporadic, 1; very mild infiltration, sporadic, 2; mild infiltration, sporadic distribution, 3; moderate infiltration including 0–2 subepithelial clusters, 4; moderate infiltration, moderate numbers of subepithelial clusters (>2), 5; massive infiltration, massive numbers of supepithelial clusters (>10). All evaluations and scoring was performed completely blinded to mouse number, treatment group and infection status.

### Statistical analysis

IFU counts among groups were analyzed by a Kruskal-Wallis test followed by Dunn’s multiple comparisons test, if more than two groups were compared, or by a Mann-Whitney test if two groups were compared, as indicated in the figure legend. Cell percentages among groups were analyzed by a one-way ANOVA followed by Tukey’s multiple comparison test if more than two groups were compared, or by a two-sided unpaired two-sample *t*-test if two groups were compared, as indicated in the figure legend. Prism version 7 software (GraphPad) was used for analysis. A *p*-value of ≤ 0.05 was considered as a significant difference.

### Reporting summary

Further information on research design is available in the Nature Research Reporting Summary linked to this article.

## Supplementary information

Supplementary Information

Reporting summary

## Data Availability

The data that support the findings of this study are available from the corresponding author upon reasonable request.
