# Supplementary material for: Parenteral vaccination protects against transcervical infection with Chlamydia trachomatis and generate tissue-resident T cells post-challenge
Source: NPJ Vaccines. 2020 Jan 23;5:7. doi: 10.1038/s41541-020-0157-x (PMC6978417; doi:10.1038/s41541-020-0157-x)
Supplement: Supplementary file 1 — Supplementary Information [file 41541_2020_157_MOESM1_ESM.pdf]

Parenteral vaccination protect against a transcervical infection with *Chlamydia trachomatis* and generate tissue resident T cells post challenge

Nina Dieu Nhien Tran Nguyen[1], Anja W. Olsen[1], Emma Lorenzen[1], Peter Andersen[1], Malene Hvid[2], Frank Follmann[1], Jes Dietrich[1,]*

[1] Statens Serum Institut, Department for Infectious Disease Immunology, Denmark
[2] Department of Biomedicine and Department of Clinical Medicine, Aarhus University

Supplementary information

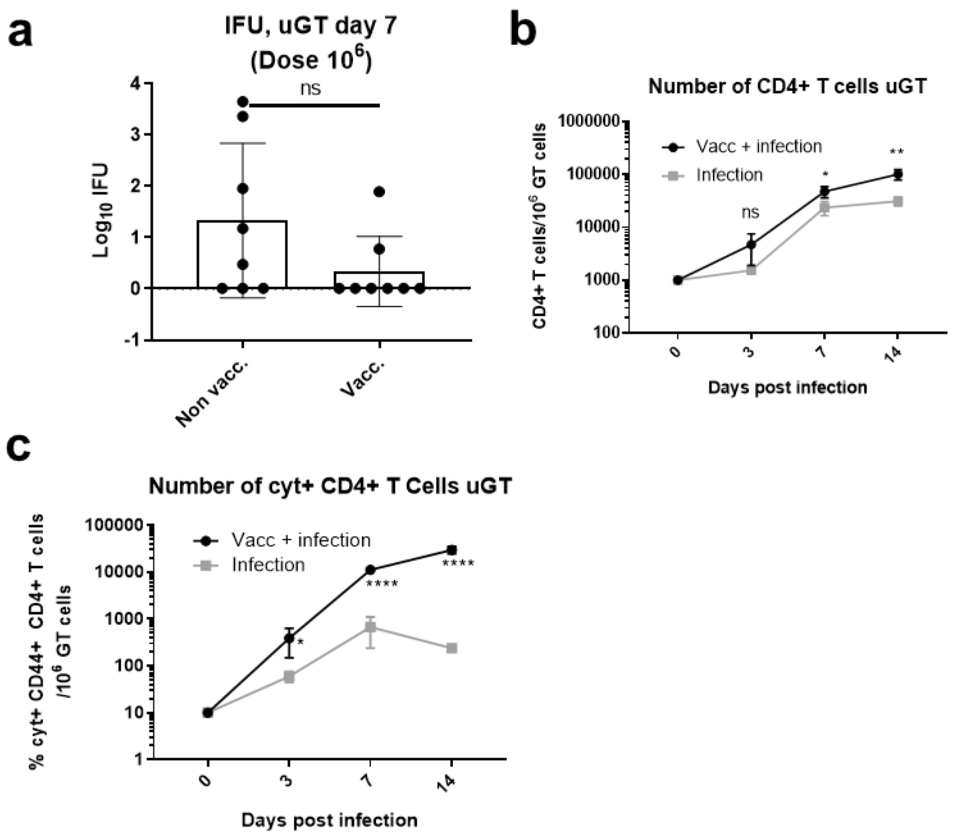

<u>Supplementary figure 1.</u>

T cells are recruited to uGT upon infection and responds, but no effect on bacterial level is observed.

Groups of female B6C3F1 mice (n=8) were vaccinated three time subcutaneously (s.c.) with CTH522 antigen and CAF01 adjuvant at two weeks intervals. Three weeks after the last immunization, the mice received a transcervical (TC). 7 days post a TC infection bacterial burden was determined by counting IFUs in upper GT (uGT) swab samples from mice receiving a dose of 10^6 *C.t.* SvD. The experiment was part of the experiment shown in figure 2 a and b **a.** Bars indicate medians with interquantile ranges (IQR). Statistical significance was evaluated by a Mann Whitney test using GraphPad Prism version 7.04. The number of CD4+ T cells in the GTs are shown in mice receiving 10^5 IFU of *C.t.* SvD **b.** The number of cyt+ CD4+ T cells (expressing any combination of the cytokines INFγ/IL-2/IL-17/TNFα) in uGT at the indicated timepoint post infection **c**. Statistical significance was evaluated by an unpaired *t* test using GraphPad Prism version 7.04. ns: not significantly different. *p<0.05, **p<0.01, ****p<0.0001.

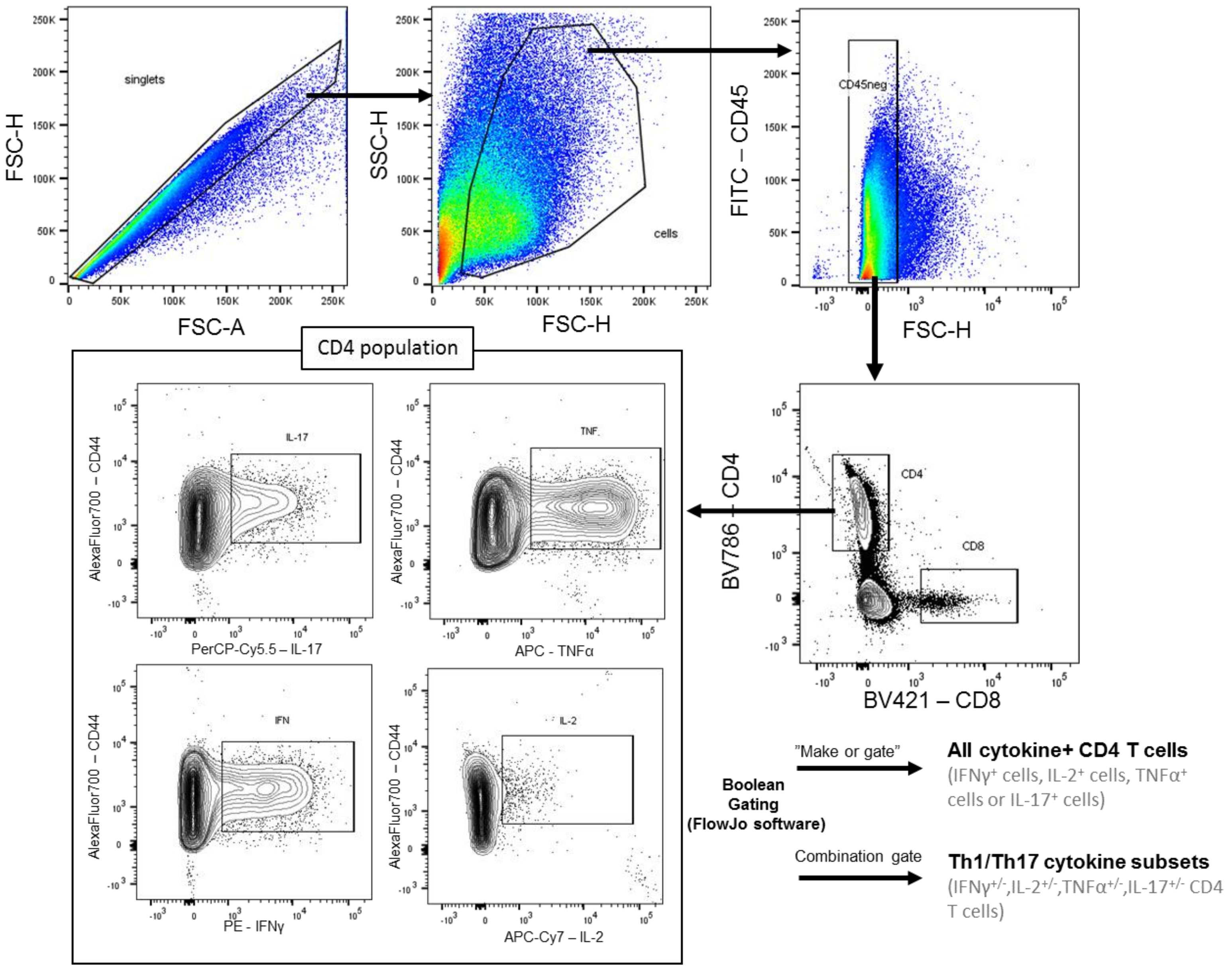

Supplementary figure 2.

Flow cytometry gating strategy for T cell analysis Gating strategy used to analyze CD4 T cells, CD8 T cells, cytokine+ CD4 T cells and Th1/Th17 cells in uGT and ILN samples. Doublets were excluded by plotting FSC-height (H) vs. FSC-area (A). Cell debris were excluded on SSC-H and SSC-A. To analyse tissue leukocytes and exclude vascular leukocytes the mice were i.v.- injected with CD45-FITC before euthanization and i.v. $CD45^{+}$ cells were excluded from the analysis. CD4 and CD8 gating was applied and CD4 cells were further analysed for $CD44^{+}$ $cytokine^{+}$ (IFNγ,IL-2,TNFα,IL-17) expression. To determine the frequency of all $cytokine^{+}$ CD4 T cells and Th1/Th17 cytokine subsets boolean gating was applied to the gated $IFN\gamma^{+}$ cells, $IL\text{-}2^{+}$ cells, $TNF\alpha^{+}$ cells, $IL\text{-}17^{+}$ cells. To determine the frequency of the cyt+ CD4 T cell population all the four $CD44^{+}$ cyt^{+} CD4 T populations were included in one gate by the "make or gate" function in FlowJo software. The frequency of Th1/Th17 cytokine subsets were determined by creating combination gates of all the possible combinations of the four $CD44^{+}$ cyt^{+} population ($CD44^{+}$, $IFN\gamma+^{/-}$,$IL\text{-}2^{+/-}$,$TNF\alpha^{+/-}$,$IL\text{-}17^{+/-}$ CD4 T cells)

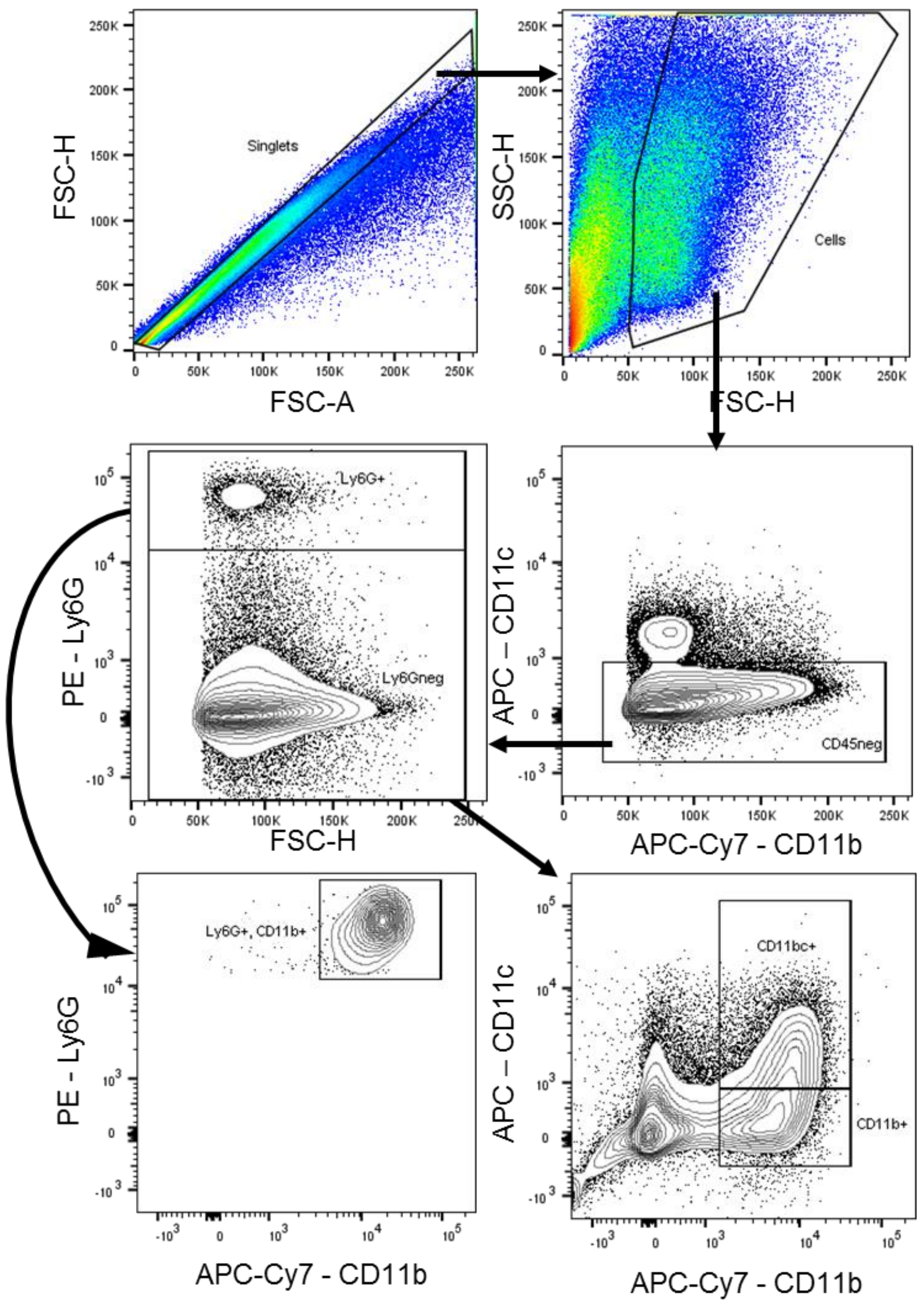

<u>Supplementary figure 3.</u>

Flow cytometry gating strategy for innate cell analysis.

Gating strategy used to analyze in uGT and ILN samples. Doublets were excluded by plotting FSC-height (H) vs. FSC-area (A). Cell debris were excluded on SSC-H and SSC-A. To analyse tissue leukocytes and exclude vascular leukocytes the mice were i.v.- injected with CD45-FITC before euthanization and i.v. $CD45^+$ cells were excluded from the analysis. Cells were then distinguished into $Ly6G^{-/+}$ populations. $Ly6G^+$ cells were further gated for $Ly6G^+$ $CD11b^+$ cells to determine the neutrophil population. The $Ly6G^-$ population were gated into macrophages ($Ly6G^-CD11b^+CD11c^-$) and dendritic cells (DCs)($Ly6G^-CD11b^+CD11c^+$).

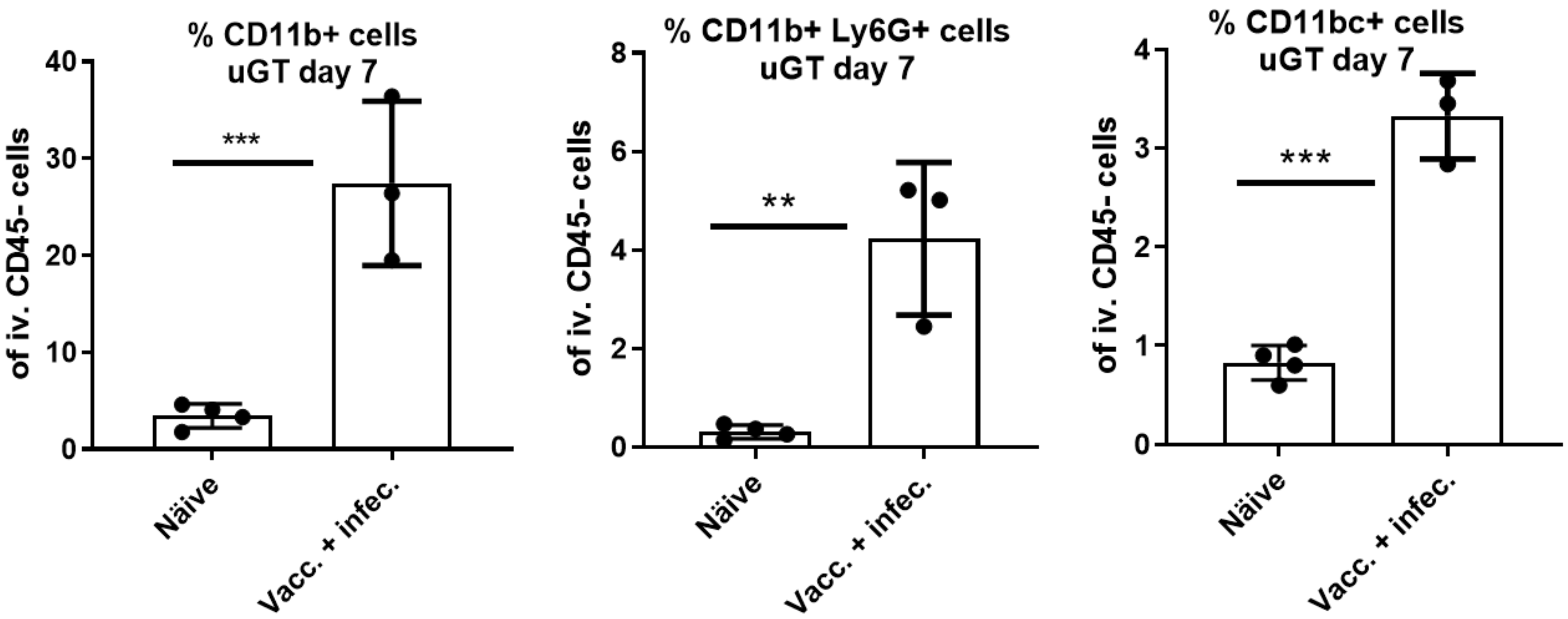

Supplementary figure 4.

Innate response towards an infection with 10^5 IFU of *C.t.* SvD.

Groups of female B6C3F1 mice received a TC infection with 10^5 IFU of *C.t.* SvD. 7 days post infection, cells from the uGT were stained with the indicated surface markers and analyzed by flow cytometry for the percentage of innate cells in uGT. Neutrophils ($Ly6G^+CD11b^+$), macrophages ($Ly6G\text{-}CD11b^+CD11c^-$) and dendritic cells ($Ly6G^-CD11b^+CD11c^+$). Bars indicate means ±SD, **p<0.01, ***p<0.001.

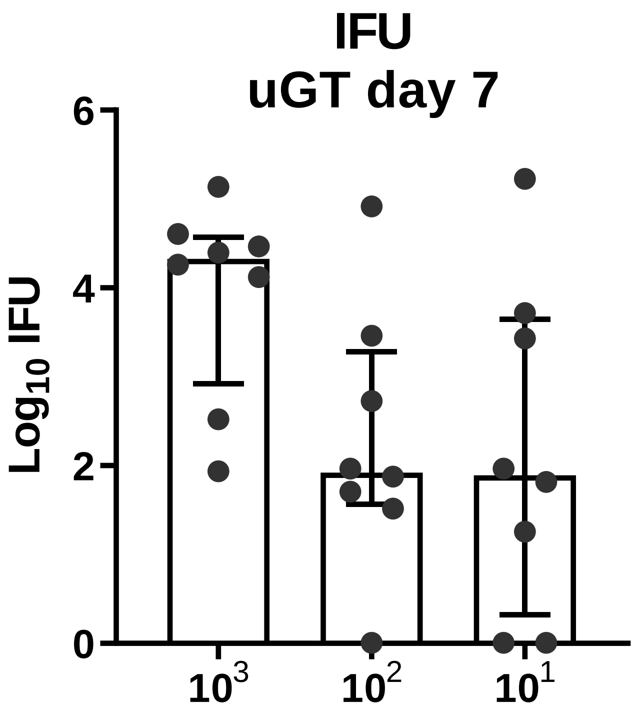

<u>Supplementary figure 5.</u>

Bacterial burden 7 days post infection at low infectious doses.

Bacterial burden (IFU) were determined 7 days post infection in uGT from groups of female B6C3F1 mice (n=8) who received a TC infection with 10^3, 10^2 or 10^1 IFU of *C.t.* SvD. Bars indicate medians with IQR.

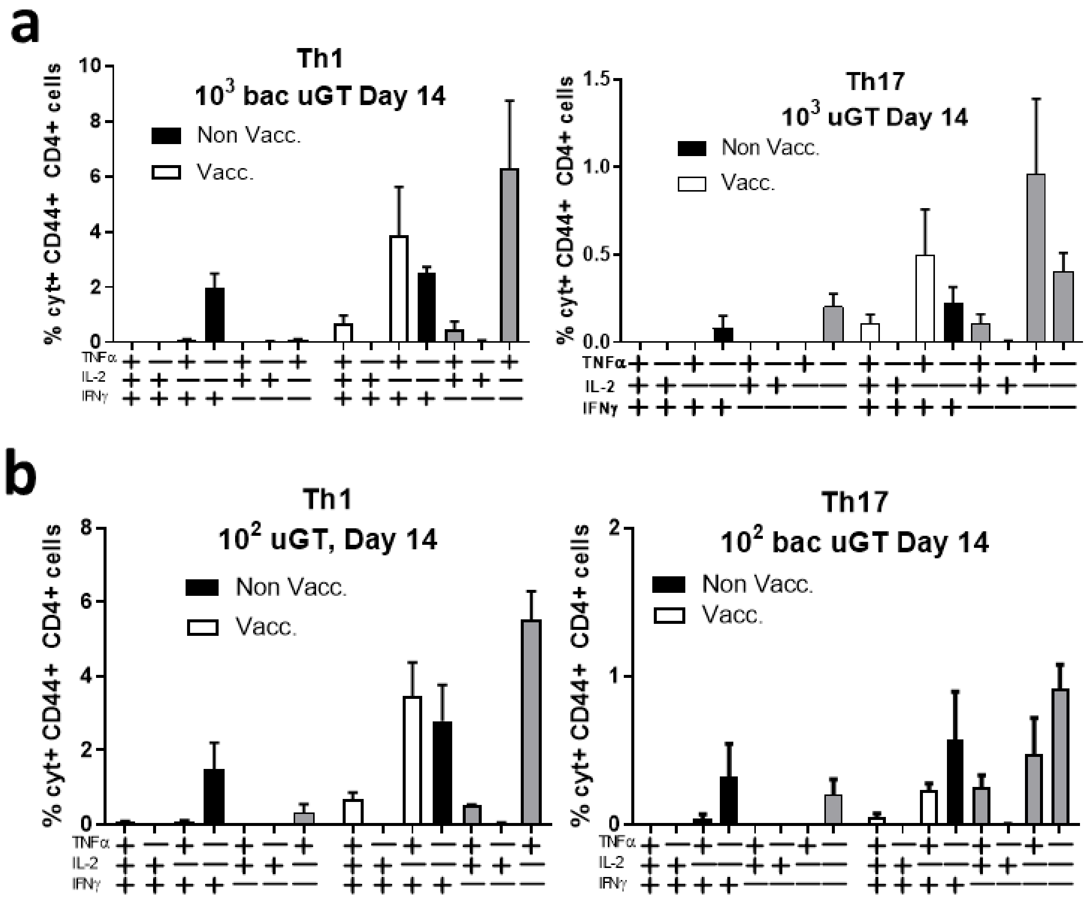

<u>Supplementary figure 6.</u>

Vaccination prevent infection driven low quality Th1 and TH17 T cell cytokine subsets

Groups of female B6C3F1 mice (n=8) were vaccinated three time s.c. with CTH522 antigen and CAF01 adjuvant with two weeks intervals. Three weeks after the last vaccination mice were infected with 10^3 **a**, or 10^2 IFU **b**, of *C.t.* SvD. 14 days post infection T cells in uGT were stained for indicated intracellular cytokines. IL-17 negative CD4 T cells (Th1) and IL-17 positive CD4 T cells (Th17) in the uGT were analyzed for cytokine subsets by flow cytometry. Grey bars indicate resting T cell subsets (expressing IL2 and/or TNFα), white bars indicate effector subsets that also express INFγ and black bars indicate subsets expressing only IFNγ within the Th1 and Th17 population. Bars indicate means ±SD

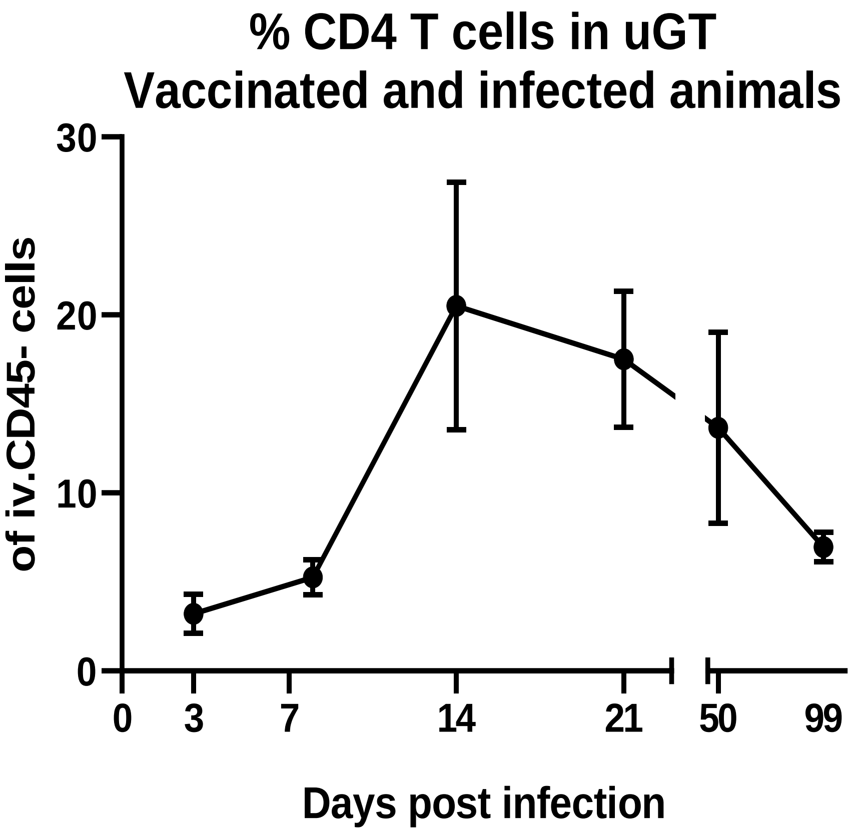

Supplementary figure 7.

Percentage of CD4+ T cells in upper genital tract and their cytokine expression.

Groups of female B6C3F1 mice (n=8) were vaccinated three time s.c. with CTH522 antigen and CAF01 adjuvant with two weeks intervals. Three weeks after the last immunization, the mice received a TC infection with 10^3 IFUs of *C.t.* SvD. Cells from uGT were surface stained for CD4 and analyzed by flow cytometry to examine the percentage of CD4+ cells in uGT at the indicated days post infection. Graph show the means ±SD.

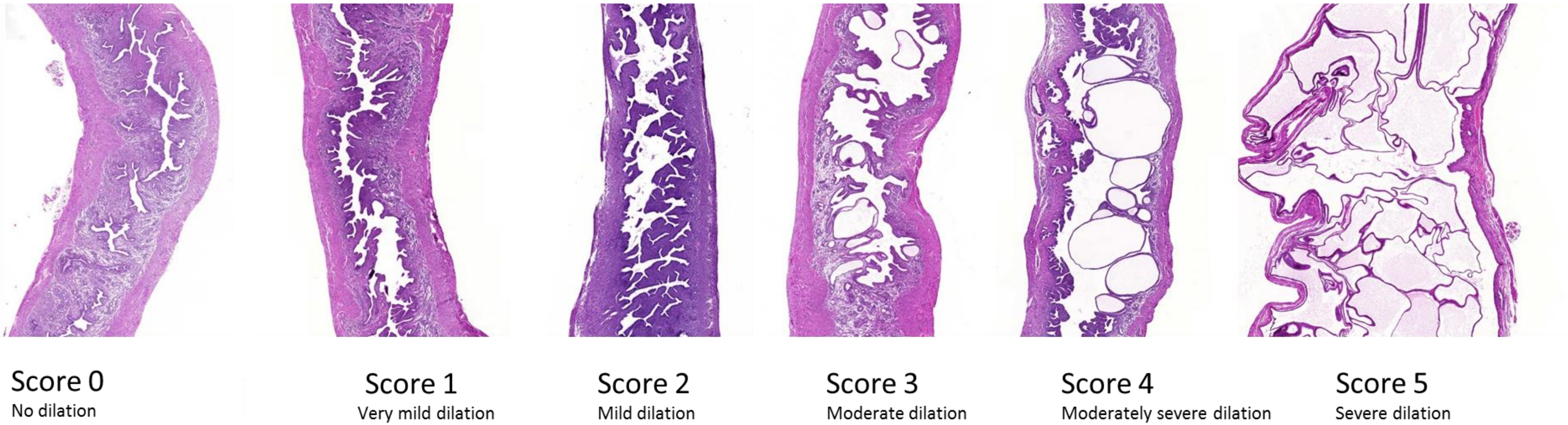

Supplementary fig. 8

Pathology score.

Examples of the histopathological scoring based on the degree of uterine horn hydrometra after an infection with 10^3 *C.t.* SvD. H&E stained tissue sections were assigned an inflammatory score according to the degree of hydrometra/uterine lumen dilation and glandular duct dilation. The following scores were used: 0; no dilation, 1; very mild dilation, 2; mild dilation, 3; moderate dilation with glandular duct dilation, 4; moderately severe dilation and moderate glandular duct dilation, 5; severe dilation and severe glandular duct dilation.

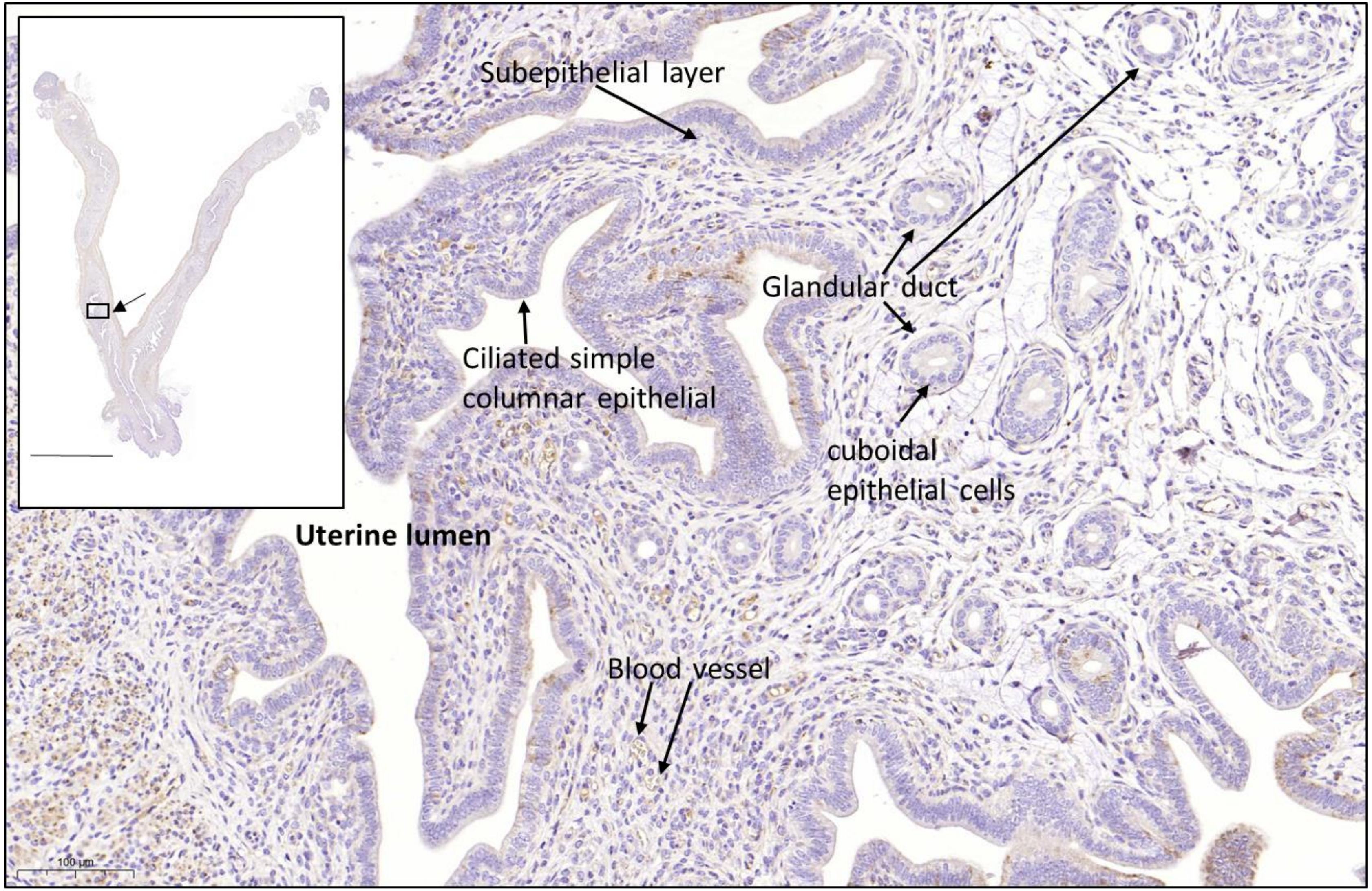

Naive mouse

Supplementary fig. 9
Immunohistochemical staining of CD4 and CD8 cells in the GT of näive mice.

GTs from näive female B6C3F1 mice (n=3) were fixed in 4 % formaldehyde and afterwards embedded in paraffin, sectioned and stained to detect CD8 (DAB chromogen - brown) and CD4 (Permanent red chromogen) epitopes. The sections were counterstained with Mayer Hematoxylin.

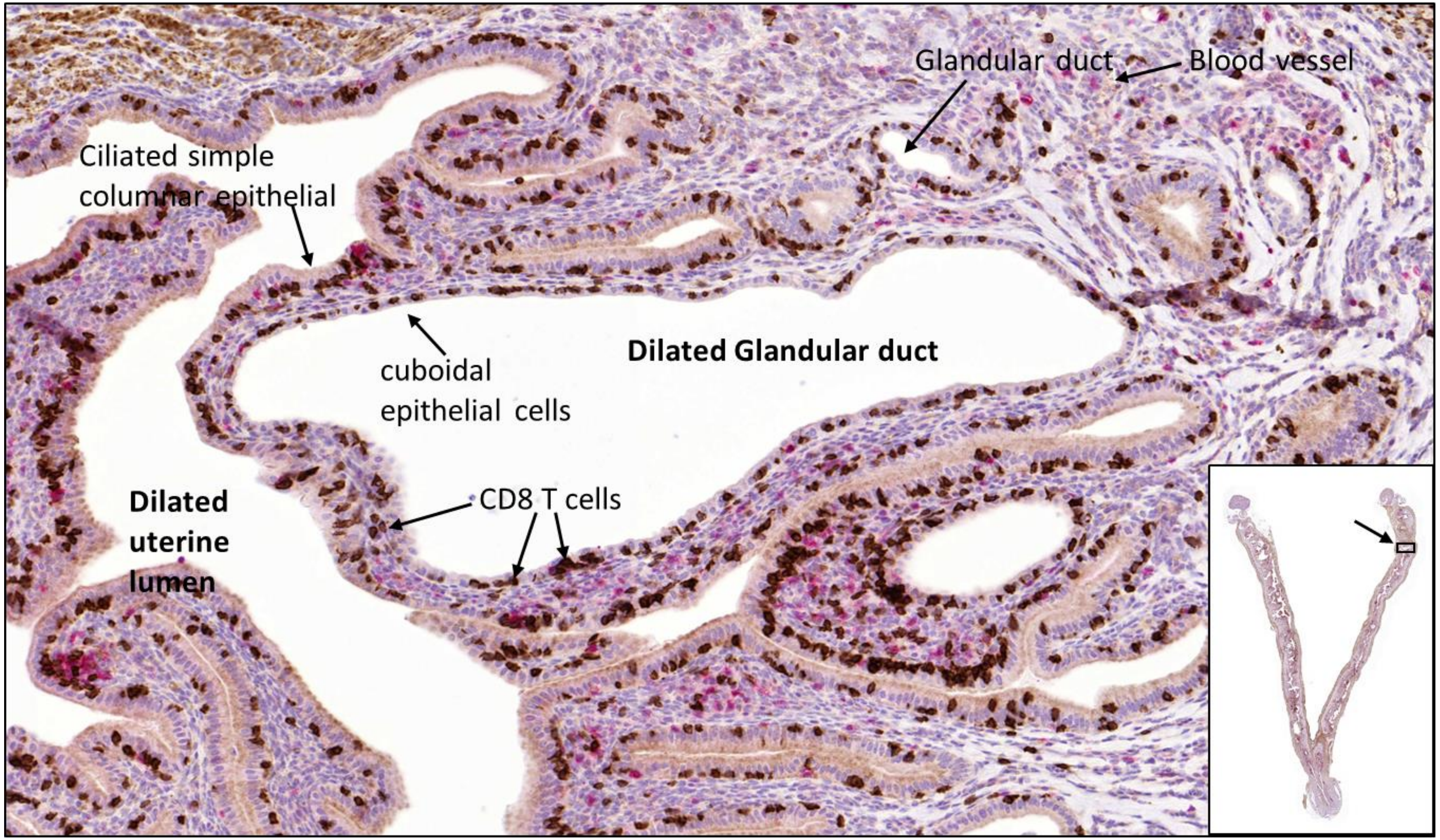

Non-vaccinated and infected mouse

<u>Supplementary fig. 10</u>
Immunohistochemical staining of CD4 and CD8 cells in the GT of non-vaccinated and infected mice.
GTs from non-vaccinated and 10^3 *C.t.* SvD infected female B6C3F1 mice (n=12) were fixed in 4 % formaldehyde and afterwards embedded in paraffin, sectioned and stained to detect CD8 (DAB chromogen - brown) and CD4 (Permanent red chromogen) epitopes. The sections were counterstained with Mayer Hematoxylin.

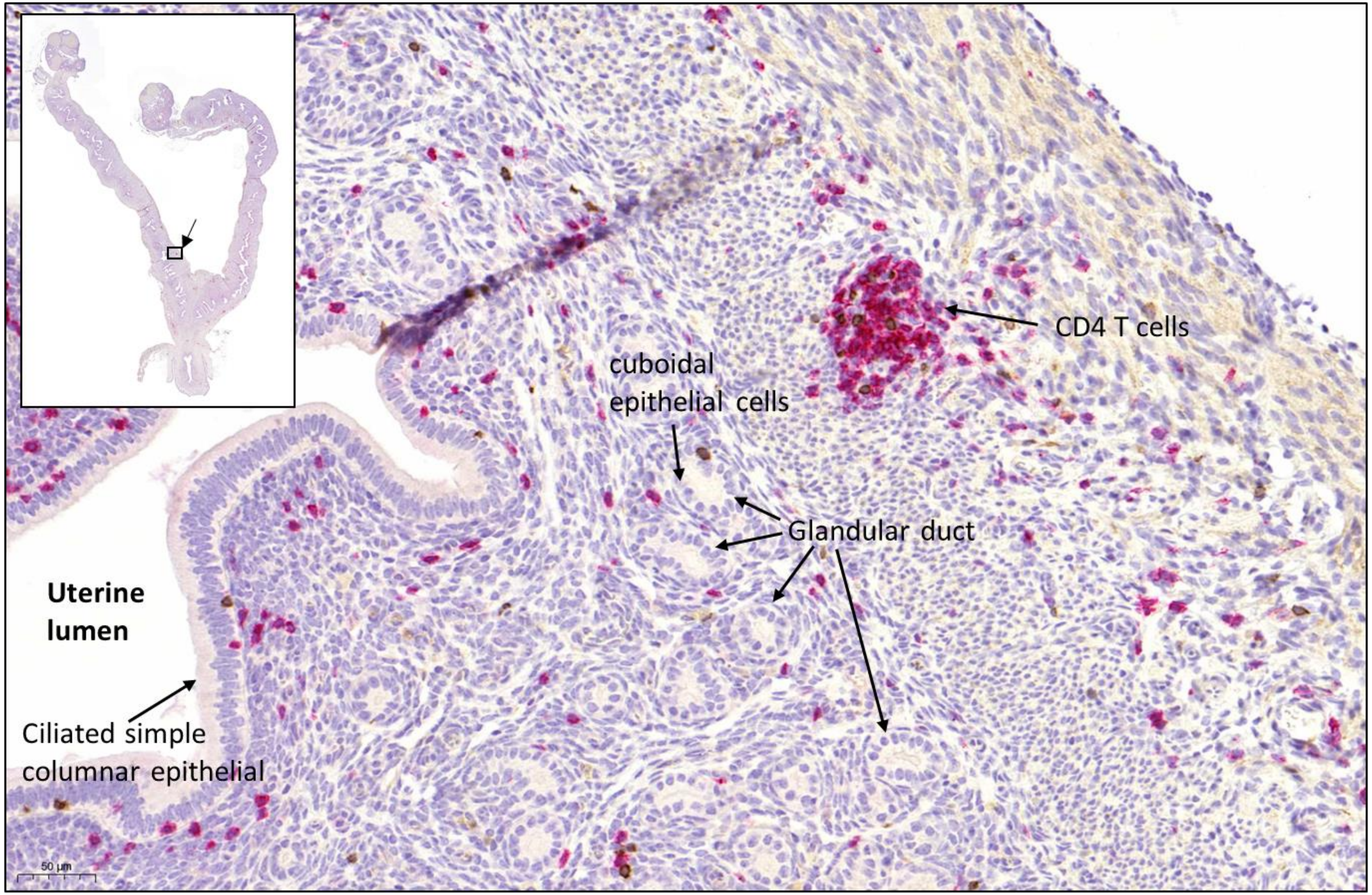

Vaccinated and infected mice

Supplementary fig. 11

Immunohistochemical staining of CD4 and CD8 cells in the GT of vaccinated and infected mice. Female B6C3F1 mice (n=8) were vaccinated three time s.c. with CTH522 antigen and CAF01 adjuvant with two weeks intervals. Three weeks after the last immunization, the mice received a TC infection with 10^3 *C.t.* SvD. GTs were harvested, fixed in 4 % formaldehyde and afterwards embedded in paraffin, sectioned and stained to detect CD8 (DAB chromogen - brown) and CD4 (Permanent red chromogen) epitopes. The sections were counterstained with Mayer Hematoxylin.
